# Glioblastoma and cerebral organoids: development and analysis of an *in vitro* model for glioblastoma migration

**DOI:** 10.1002/1878-0261.13389

**Published:** 2023-02-18

**Authors:** Veronika Fedorova, Veronika Pospisilova, Tereza Vanova, Katerina Amruz Cerna, Pavel Abaffy, Jiri Sedmik, Jan Raska, Simona Vochyanova, Zuzana Matusova, Jana Houserova, Lukas Valihrach, Zdenek Hodny, Dasa Bohaciakova

**Affiliations:** ^1^ Department of Histology and Embryology, Faculty of Medicine Masaryk University Brno Czech Republic; ^2^ International Clinical Research Center (ICRC) St. Anne's University Hospital Brno Czech Republic; ^3^ Laboratory of Gene Expression Institute of Biotechnology CAS, BIOCEV Vestec Czech Republic; ^4^ Department of Genome Integrity Institute of Molecular Genetics of the Czech Academy of Sciences Prague Czech Republic

**Keywords:** cerebral organoids, GLICO, glioblastoma, induced pluripotent stem cells

## Abstract

It is currently challenging to adequately model the growth and migration of glioblastoma using two‐dimensional (2D) *in vitro* culture systems as they quickly lose the original, patient‐specific identity and heterogeneity. However, with the advent of three‐dimensional (3D) cell cultures and human‐induced pluripotent stem cell (iPSC)‐derived cerebral organoids (COs), studies demonstrate that the glioblastoma‐CO (GLICO) coculture model helps to preserve the phenotype of the patient‐specific tissue. Here, we aimed to set up such a model using mature COs and develop a pipeline for subsequent analysis of cocultured glioblastoma. Our data demonstrate that the growth and migration of the glioblastoma cell line within the mature COs are significantly increased in the presence of extracellular matrix proteins, shortening the time needed for glioblastoma to initiate migration. We also describe in detail the method for the visualization and quantification of these migrating cells within the GLICO model. Lastly, we show that this coculture model (and the human brain‐like microenvironment) can significantly transform the gene expression profile of the established U87 glioblastoma cell line into proneural and classical glioblastoma cell types.

## Graphical abstract

Modeling glioblastoma *in vitro* remains a major challenge. This study established a protocol for coculturing cerebral organoids and glioblastoma spheroids that allows studying the glioblastoma under conditions resembling *in vivo* state. Approaches for visualization and evaluation of glioblastoma cell migration were introduced. Importantly, mRNA‐seq showed that glioblastoma cells cocultured with cerebral organoids changed their gene expression toward neural cell identity.

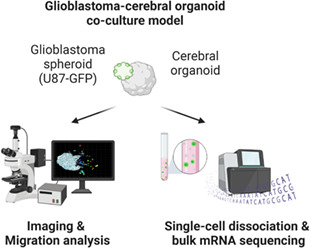

**Abbreviations**
COs: cerebral organoids
DEGs: differentially expressed genes
ECM: extracellular matrix
GLICO: glioblastoma‐cerebral organoid coculture model
GSCs: glioblastoma stem cells
GSEA: gene set enrichment analysis
iPSCs: induced pluripotent stem cells
ORA: over‐representation analysis
PDXs: patient‐derived xenografts
U87: U‐87 MG glioblastoma cell line

## 1 Introduction

Glioblastoma (grade IV astrocytoma) is the most aggressive and lethal form of brain tumors with extremely poor prognostic outcome despite intensive therapy. The median survival of adults with glioblastoma after surgical resection, radio‐, and chemotherapy with DNA alkylating agent temozolomide is currently 14 months, and the 5‐year survival rate is less than 5% [1, 2]. Intriguingly, this 5‐year survival remains poor, with no apparent improvement over the past decade [3], indicating the need for better treatment strategies resulting from improved models and new analytical approaches to studying glioblastoma biology and invasiveness.

It is challenging to adequately model the growth and migration of patient‐derived glioblastoma *in vitro*. To this date, numerous reports have shown that upon transferring the resected tumor tissue to a 2D cell culture environment, glioblastoma cells lose their original (patient‐specific) identity and heterogeneity. Even after maintaining glioblastoma tissue in serum‐free cell culture conditions or selecting glioblastoma stem cells (GSCs), 2D cell culture invariably generates cell populations markedly different from the original tumor. Additionally, the intrinsically invasive behavior of glioblastoma cannot be modeled in 2D culture, as it requires 3D brain(‐like) tissue. Therefore, studies thus far have essentially focused on generating patient‐derived xenografts (PDXs) using mice models. Although they have brought significant improvement in the maintenance of glioblastoma *ex vivo*, they remained challenging to work with and inadequate for simulating specifically the human brain environment. Since the advent of 3D cell cultures, glioma spheres and tumor organoids have become increasingly used. Additionally, since the description of cerebral organoids (COs) derived from induced pluripotent stem cells (iPSCs) [4], several reports recently demonstrated that COs could indeed be used for modeling glioblastoma *in vitro*. As they faithfully recapitulate the developing human brain, they can either be genetically modified to develop glioblastoma [5, 6] or used as a scaffold for supporting the growth of patient‐derived glioblastoma tissue [7, 8, 9, 10, 11, 12]. Importantly, these studies are increasingly demonstrating that the glioblastoma‐cerebral organoid (GLICO) coculture model is superior to other glioblastoma culture systems, likely because the COs provide the glioblastoma with the appropriate microenvironment [12]. However, with their increasing use, reports thus far have employed a plethora of approaches toward generating GLICO cocultures (reviewed in [13]) and described a different ability and the time needed for gliomas to migrate within the COs. Thus, despite all recent advances, it remains challenging to recapitulate some aspects of the GLICO coculture system.

Prompted by the increasing number of studies reporting the GLICO model as superior to other glioblastoma culture systems, we set out to optimize this coculture system in our hands. Importantly, we aimed to implement several crucial parameters that were lacking or not sufficiently specified in the previous reports, namely: (a) the use of adequately mature COs for co‐culture with glioblastoma; (b) the absence of additional extracellular matrix (ECM) scaffolds/proteins (e.g., Matrigel); (c) the development of visualization approach that would allow us to identify and quantify the extent of migration within COs; and (d) optimization of isolation of glioblastoma cells from coculture model with subsequent proof‐of‐concept molecular analytics. As a glioblastoma model, we used a well‐established, fluorescently labeled U‐87 MG glioblastoma cell line (further referred to as U87). Our results indicate that (55‐day‐old) mature COs support the growth of U87 even without an ECM scaffold, however, with a relatively long time needed to initiate cell migration inside the CO (≥30 days). We further show that this migration is significantly enhanced by extracellular matrix proteins, such as Matrigel or Geltrex, with Matrigel also having a substantial impact on glioblastoma cell growth. We also report on developing a strategy for visualization and quantification of glioblastoma cells within COs using confocal microscopy and tissue clearing that can be adapted for the analysis and quantification of glioblastoma cell migration. Lastly, we optimized the dissociation of COs into single cells and, upon FACS sorting of fluorescently labeled glioblastoma cells, performed bulk mRNA sequencing. Data show that 40 days of the coculture of established U87 glioblastoma cell line in human brain organoids significantly change their gene expression toward neural cell identity and induce a switch toward proneural and classical glioblastoma cell type. Altogether, our results not only point to the differences between GLICO culture methods and a significant effect of ECM proteins on enhanced tumor growth but also bring evidence that mimicking the human brain microenvironment using COs is a powerful technique for studying gliomas under conditions that better represent the *in vivo* conditions.

## 2 Materials and methods

### 2.1 Culture of induced pluripotent stem cells

Three independent human iPSC lines ‐ MUNIi008‐A, MUNIi009‐A, and MUNIi010‐A (RRID:CVCL_A4PG, RRID:CVCL_A4PH, and RRID:CVCL_A4PI) derived and characterized in our laboratory were used to generate cerebral organoids [14]. The authentication process of these cell lines is also explained in [14]. As described previously [14, 15], iPSCs were routinely screened for mycoplasma, grown in feeder‐free conditions on Matrigel‐coated dishes (Corning, Corning, NY, USA) in mTeSR™1 medium (STEMCELL Technologies, Vancouver, Canada) and passaged using 0.5 mm EDTA (Thermo Fisher Scientific, Waltham, MA, USA) in PBS or manually. Primary human fibroblasts for the generation of iPSCs were obtained from Corriel Institute based on NIGMS human genetic cell repository Material Transfer Agreement.

### 2.2 Cerebral organoid differentiation and preparation of thick organoid sections

With a few modifications, the generation of COs followed the protocol described in [16, 17]. Briefly, to initiate the embryoid body formation, iPSCs were detached using Accutase and plated in poly(2‐hydroxyethyl methacrylate; poly‐HEMA; Merck, Darmstadt, Germany) ‐treated nonadherent V‐shaped 96 well plates at density 2000–3000 cells per well in mTeSR™1 with 50 μm Rho‐kinase (ROCK) inhibitor Y‐27632 (Selleckchem, Houston, TX, USA). On day 2, the medium was changed for mTeSR™1 without ROCK inhibitor. When embryoid bodies were at least 400 μm in diameter, the medium was changed for the Neuroinduction medium [17]. The Neuroinduction medium was changed every day for 6 days, after which organoids were embedded in 7 μL of Geltrex™ (Thermo Fisher Scientific). Polymerized Geltrex™ droplets with organoids were detached and cultured for 10 days in Cerebral Organoid Differentiation Medium (CODM [17]) without vitamin A. After 10 days, the medium was changed for CODM with vitamin A and organoids were moved to an orbital shaker (0.035 ***g***), where they were cultured until Day 55. At this time point, they were used for GLICO model experiments. The medium was changed regularly every 2–3 days.

For experiments with thick organoid sections, COs at Day 55 were embedded in 4% agarose (Merck) and sectioned into 250 μm sections using Vibratome (Leica, Wetzlar, Germany; settings: frequency 10, speed 2). Obtained sections were then manually cleared of the agarose, placed into a CODM medium with vitamin A and used for the cocultivation with the spheroid within 24 h.

### 2.3 Preparation of fluorescently labeled glioblastoma

GBM primary cell line U87MG (RRID:CVCL_0022) was obtained from ATCC (Manassas, VA, USA) previously used in [18, 19], and prior to their use, they were screened for mycoplasma. Ectopically expressing green fluorescent protein (GFP) or tdTomato was prepared by stable transfection using linearized pEGFP‐C1 vector or pcDNA3.1(+)/Luc2 = tdT (Addgene, Watertown, MA, USA), respectively, and Lipofectamine 2000 (Thermo Fisher Scientific) according to the manufacturer's recommendations. Positive cells were sorted using FACS.

### 2.4 U87‐GFP/tdTomato cell culture and spheroid formation

U87‐GFP and tdTomato cell lines were grown on cell culture dishes in DMEM/F12 (Dulbecco's Modified Eagle Medium) with 10% FBS, 1% Glutamax, 1% nonessential amino acids, and 0.5% Penicillin/Streptomycin and passaged using TrypLE (all from Thermo Fisher Scientific). To generate spheroids, cells were detached using TrypLE, counted, and seeded at the density of 2000 cells per well in a poly‐HEMA‐treated nonadherent V‐shaped 96 well plate. The plate was centrifuged at 200 ***g***, 2 min to facilitate spheroid formation. Fluorescently labeled U87 spheroids were used for GLICO model experiments after 24 h.

### 2.5 GLICO coculture on an inclined plane and with ECM protein scaffolds

For setting up a GLICO model without ECM protein scaffolds, we used poly‐HEMA‐treated nonadherent 12 well plates with CODM with vitamin A. A single U87‐GFP spheroid was placed on the bottom edge of each well. Subsequently, a single CO was positioned on the top of the spheroid. One side of the 12 well plate was then placed on top of an empty culture dish at an angle of 45° resulting in an inclined plane. This inclination ensured the attachment of the glioblastoma spheroid and the CO. The GLICO was allowed to form for 48 h in the inclined plate. After 48 h, the attachment of the spheroid was examined. If the spheroid had not attached to the CO, both the spheroid and the CO were repositioned and allowed to attach for another 24 h in the inclined plate. Subsequently, the plate was kept in an incubator without shaking, and half of the volume of the medium was changed carefully every 2 days, making sure not to separate the GLICO apart. After 4 days, the plate was moved to an orbital shaker (57 r.p.m.) and cultured until the analysis day. The medium was changed three times a week.

To create the GLICO model with ECM protein scaffolds, a single U87‐GFP spheroid was placed in the vicinity of an intact CO or the thick section in an empty cell culture dish. The two components were embedded in a 10 μL droplet of Matrigel™ or Geltrex™, ensuring the spheroid and the intact CO or thick section remained in close contact and incubated for 10 min at 37 °C until Matrigel™ or Geltrex™ polymerized. The polymerized droplet with GLICO was gently transferred into nonadherent cell culture dishes using sterile spoons and cultured in CODM with vitamin A on an orbital shaker (0.035 ***g***) until the day of analysis. The medium was changed three times a week.

### 2.6 Western blotting

Western blotting was performed as previously described in [20]. Briefly, samples were lysed in 1% SDS lysis buffer (50 mm TRIS–HCl, pH 6.8, 1% SDS, 10% glycerol) and sonicated. Protein concentration was measured using DC™ Protein Assay (Bio‐Rad, Hercules, CA, USA) and adjusted to 1 μg·μL^−1^. Samples were mixed with bromphenol blue solution (0.2% bromphenol blue, 10% 2‐mercaptoethanol) and denatured at 95 °C for 6 min. Proteins were then loaded on a 10% polyacrylamide gel, separated by SDS/PAGE, and transferred onto PVDF membranes (Merck). Membranes were blocked in 5% milk (pH 7.4) and incubated with primary antibodies at 4 °C overnight. The next day, membranes were washed with TBS‐Tween‐20, incubated with secondary antibodies and ECL solution, and imaged using Chemidoc (Bio‐Rad). All antibodies used for western blotting are listed in Table S1.

### 2.7 Histological preparation and whole‐mount of organoid samples

Before all immunostaining methods, harvested GLICOs were fixed with 3.7% formaldehyde for 1 h. They were then washed with PBS two times and stored in sterile PBS at +4 °C until processing.

#### 2.7.1 Immunohistochemistry (IHC)

Fixed GLICO was embedded in 3% agarose (Merck), followed by paraffin, and sectioned in 2 μm thin sections. The sections were deparaffinized in xylene, rehydrated in descending ethanol series (96–80–70–50%), and recovered in antigen retrieval (pH 6, DAKO, Carpinteria, CA, USA) for 20 min per 98 °C. The sections were then permeabilized in 0.2% Triton‐X‐100 (Merck) in PBS and incubated with primary antibodies at 4 °C overnight. The next day, the sections were washed multiple times with PBS and incubated with secondary antibodies. Nuclei were visualized by Hoechst 33342 (Thermo Fisher Scientific). Sections were mounted using Mowiol (Mowiol 4–88, P‐Lab, Prague, Czech Republic) and imaged using confocal microscopy (Zeiss Axio Observer.Z1 with confocal unit LSM 800; Zeiss, Oberkochen, Germany). All antibodies used for immunohistochemistry are listed in Table S2.

#### 2.7.2 Whole‐mount and CUBIC clearing

Fixed organoids were incubated in a CUBIC1 reagent at 37 °C for 5–7 days with one CUBIC1 reagent exchange (after 3 days) [21]. After incubation, samples were washed and incubated with Hoechst 33342 (Thermo Fisher Scientific) at 4 °C for 24 h. The GFP and tdTomato signal of glioblastoma cells was stable and did not need to be enhanced using specific antibodies. Next, samples were washed and incubated in a CUBIC2 reagent at room temperature for 24–36 h. All incubations were done with gentle shaking. Cleared and stained organoids were embedded in a mounting solution in μ‐Slide 8 Well (IBIDI) for confocal microscopy (Zeiss Axio Observer.Z1 with confocal unit LSM 800; Zeiss). Whole‐mount and CUBIC clearing buffer compositions are listed in Table S3.

### 2.8 Microscopy

The histological sections were imaged with the inverted microscope Zeiss Axio Observer.Z1 with confocal unit LSM 800, equipped with solid‐state lasers (405, 561, and 640 nm) and Plan‐Neofluar 10×/0.30 AIR and Plan‐Neofluar 20×/0.50 AIR objectives using zen blue software (Zeiss). Images with 0.329 × 0.329 × 5.500 μm (10×) and 0.156 × 0.156 × 0.700 μm (20×) pixel size were acquired using GaAsP PMT detectors. The acquisition parameters for Alexa Fluor 405, 568, and 647 were: 410–470, 565–617, and 656–700 nm (emission wavelength range) and 1.03 μs (pixel dwell time). The pinhole was set to 1 AU–8.2 μm (10×) and 1 μm (20×). Line average of 2 was applied to all channels.

Cleared whole‐mount organoids were imaged with the inverted microscope Zeiss Axio Observer.Z1 with confocal unit LSM 800, equipped with solid‐state lasers (405, 488, and 561 nm) and Plan‐Neofluar 5×/0.16 AIR and Plan‐Neofluar 10×/0.30 AIR objective using zen blue software (Zeiss). Images were acquired using GaAsP PMT detectors. The acquisition parameters for Hoechst, GFP, and tdTomato were: 400–486, 486–558, and 575–700 nm (emission wavelength range) and 1 μs (pixel dwell time). The Line average of 2 was applied to both channels. The pinhole was set to 1 AU 32 μm (5×) and 8.2 μm (10×). For z‐stack imaging, slices were acquired with a 16 μm (5×) and 4.1 μm (10×) z‐step size.

### 2.9 Analysis of glioblastoma cell migration

To evaluate glioblastoma cell migration, we used commercially available software imaris version 9.8.2 (Bitplane, South Windsor, CT, USA). Detection of individual glioblastoma cells was performed in imaris using the ‘Surface’ module. For individual glioblastoma cell detection, the smooth parameter was set to 0.8 μm. Thresholding was based on the background subtraction with the largest sphere diameter of 5 μm and manual correction of the intensity of each sample. For splitting touching objects, the seed point diameter was set to 7 μm. Droplet number was filtered using a quality filter, setting a droplet threshold manually for each sample. Finally, the samples were evaluated and, if necessary, the individual cells were split manually. Manual corrections of the automatic counts were performed to ensure that each migrated glioblastoma cell was appropriately outlined. Subsequently, the boundary between the glioblastoma sphere and the migrating glioblastoma cells was determined manually. The estimated parameters included (a) the number of glioblastoma cells that migrated from the glioblastoma sphere within the cerebral organoid, and (b) the shortest distance of migrated glioblastoma cells to the surface of the glioblastoma sphere. Parameters were automatically quantified using the imaris software and no selection of data with respect to the size of organoids or spheroids was performed. Data were analyzed and plotted using graphpad prism version 8 for Windows, GraphPad Software, La Jolla, CA, USA, www.graphpad.com. Three independent biological specimens of each GLICO coculture method were used to analyze glioblastoma cell migration.

### 2.10 GLICO dissociation and fluorescence‐activated cell sorting (FACS)

Before mRNA sequencing and qPCR, GLICO coculture models were dissociated, and GFP‐positive U87 glioblastoma cells were sorted using FACS. To dissociate GLICO models, whole organoids were washed in HBSS (Merck), cut into smaller pieces with a sterile scalpel, and dispersed into 125 μL per organoid of papain (Merck) dissolved in HBSS at 25 U·mL^−1^. Dissociation was performed in a shaking incubator for 10 min at 37 °C with occasional pipetting using a 1 mL pipette, followed by 10‐fold dilution with cold 2% FBS (Thermo Fischer Scientific) in HBSS and filtering through 40 μm cell strainer (Biologix, Lenexa, KS, USA). Cells were collected by centrifugation at 300 *g*, 4 °C for 5 min and resuspended in 500 μL of 2% FBS in HBSS. GFP‐positive cells were sorted using BD FACSAria™ Fusion cell sorter and lysed in RNA blue reagent (Top‐Bio, Vestec, Czech Republic) for subsequent RNA analysis. For mRNA sequencing, 40‐day‐old GLICO coculture models were used. For qPCR, 20‐ and 40‐day‐old GLICO coculture models were used.

### 2.11 RNA isolation, bulk mRNA sequencing, data processing, and analysis

For bulk mRNA sequencing, U87‐GFP spheroids were either cultured alone for 40 days (here referred to as Control) or cocultured for 40 days with 55‐day‐old COs using the inclined plane strategy (here referred to as GLICO). COs were derived from all three iPSC lines. Both control and GLICO samples were cultured in the CODM with vitamin A. After dissociation and FACS sorting, GLICO samples were harvested into RNA Blue reagent.

Total RNA was isolated from three biological replicates (with five GLICOs in each replicate) for each condition according to the manufacturer's instructions and as described previously [15]. RNA quality was assessed by TapeStation 2200 (Agilent Technologies, Santa Clara, CA, USA; 5067–5576 RNA Screen Tape), and only samples with RINe values ≥ 8.5 were used for library preparation. Poly‐A selected libraries were made from 500 ng of total RNA using QuantSeq 3'mRNA‐Seq Library Prep FWD for Illumina (Lexogen, Wien, Austria, 015.96) with i5 6 nt Unique Dual Indexing Add‐on Kit (Lexogen, 047.96) with 14–18× PCR cycles according to the manufacturer's instructions. The UMI Second Stranded Synthesis Module for QuantSeq FWD (Illumina, San Diego, CA, USA, Read 1; 081.96) was used to allow unique tagging of individual transcripts with 6 nt long Unique Molecular Identifiers (UMI). The fragment size and quality of the libraries were assessed by 5200 Fragment Analyzer System (Agilent, Santa Clara, CA, USA; DNF‐474 HS NGS Fragment Kit). The concentration of the final libraries was measured by Qubit® dsDNA HS Assay Kit (Thermo Fisher Scientific, Q32851) and sequenced with 75 bp single end reads on Illumina NextSeq® 500/550 (High Output Kit v2.5; 20024906). We successfully obtained 15–20 million reads per sample.

Data processing and analysis were performed as follows: Firstly, the 6 bp long UMIs were removed from sequences. The quality of raw reads was verified using fastqc v0.11.9 and the potential contamination was screened by fastq_screen v0.11.1 [22]. The ‘TATA’ spacer, low‐quality reads, and adaptor sequences were removed using trimmomaticse v0.36 with parameters ‘HEADCROP:4 ILLUMINACLIP: Lexogen_quantseq.fa:2:30:10 LEADING:3 TRAILING:3 SLIDINGWINDOW:4:15 MINLEN:36’. Ribosomal and mitochondrial reads were removed using sortmerna v2.1b. BAM files with alignment were created with star v2.7.0f (reference *Homo sapiens* genome version GRCh38; [23]). The count tables were generated using the script htseq‐count v0.11.4 [24] with annotation version GRCh38.87 and parameter ‘‐m union’. ENSEMBL‐IDs were used as identifiers of transcripts.

The counted data were subsequently analyzed using R‐package deseq2 v1.34.0 [25]. Rlog transformed data were processed in the principal component analysis. Differentially expressed genes (DEGs) were identified by the command ‘deseq’ with default parameters. ENSEMBL‐IDs were converted into Gene symbol using org.Hs.eg.db v3.14.0 database [26]. DEGs (adjusted *P*‐value < 0.1, log_2_FoldChange > 0.6 for upregulated, log_2_FoldChange < 0.6 for downregulated) were plotted with enhancedvolcano package v1.12.0 [27]. Functional Gene‐ontology over‐representation analysis (ORA) was conducted using clusterprofiler package v4.2.2 [28, 29] by enrichGO command with parameters “OrgDb = org.Hs.eg.db, ont = ‘ALL’, pAdjustMethod = ‘fdr’, pvalueCutoff = 0.1, qvalueCutoff = 0.2, minGSSize = 3”. Enrichment of a custom list of genes specific for different glioblastoma subtypes [30] was determined by Gene set enrichment analysis (GSEA). Custom list parameters were set to baseMean > 20. Heatmaps were constructed with complexheatmap v2.10.0 package using rlog transformed data. Final data were deposited under GEO accession number GSE216626.

### 2.12 qPCR

For protein‐coding gene expression analysis, data were collected from one experiment where GFP‐positive cells were isolated from 10–20 GLICOs and pooled for each sample. The isolated RNA was transcribed to cDNA using Transcriptor First Strand cDNA Synthesis Kit (Roche, Basel, Switzerland) according to the manufacturer's instructions, and subsequent qPCR was performed using LightCycler 480 SYBR Green I Master kit (Roche) on LightCycler 480 II (Roche). *C*_t_ values were calculated using the automated Second Derivative Maximum Method in lc480 software (Roche). The relative gene expression was calculated by normalization to glyceraldehyde 3‐phosphate dehydrogenase (*GAPDH*) expression. The mean value of the relative gene expression of all three time points was used to calculate fold change. Primers used for qPCR are listed in Table S4.

## 3 Results

### 3.1 Characterization of cerebral organoids and U87 glioblastoma cell line

**Fig. 1:**
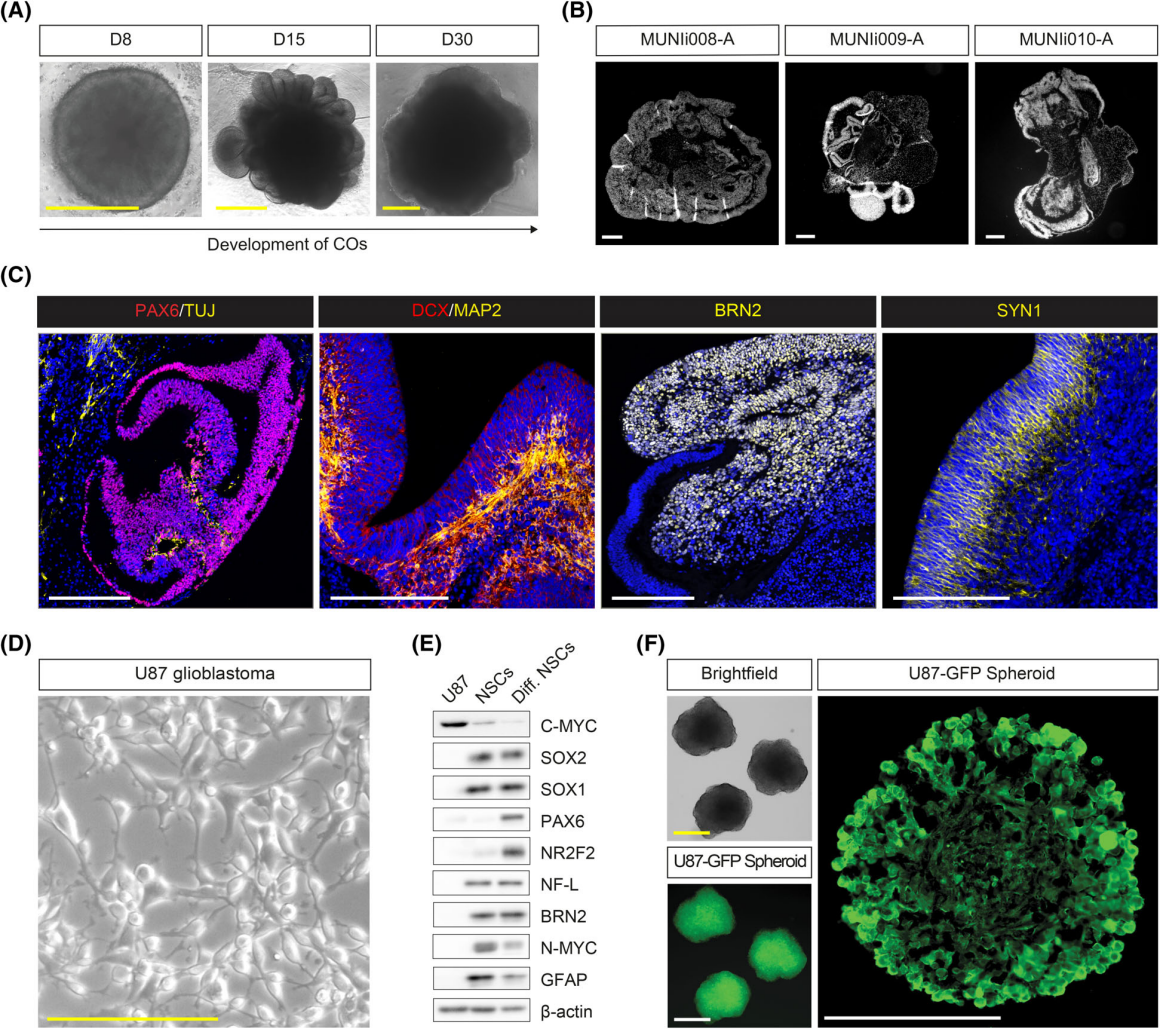
Characterization of cerebral organoids and U87 glioblastoma cell line. (A) Representative brightfield microscopy images showing the morphology of developing cerebral organoids (COs) at day 8 (D8), day 15 (D15), and day 30 (D30). Scale bar = 500 μm; *n* = 3. (B) Hoechst‐stained paraffin sections of representative images of COs derived from MUNIi008‐A, MUNIi009‐A, and MUNIi010‐A iPS cell lines showing their internal organoid organization at D55. Scale bar = 200 μm; *n* = 3. (C) Representative IHC staining images of COs showing the localization of neuronal (TUJ, MAP2, DCX, BRN2, and SYN1) and neural progenitor (PAX6) markers. Scale bar = 200 μm; *n* = 3. (D) Representative brightfield microscopy image of U87 glioblastoma cell line. Scale bar = 200 μm; *n* = 3. (E) Western blotting analysis of neuronal, glial, and neural stem/progenitor markers in U87, self‐renewing neural stem cells (NSCs), and differentiated NSCs. ß‐actin serves as loading control; *n* = 3. (F) Representative brightfield (top left) and fluorescent (bottom left) microscopy images of U87 spheroids used for coculture experiments and representative GFP^+^ image of U87 spheroid section (right). Scale bar = 200 μm; *n* = 3.

To initiate our experiments, we first characterized the two components needed to establish the GLICO model: (a) iPSCs‐derived cerebral organoids (COs) and (b) the U87 glioblastoma cell line. For the CO generation, we used three independent iPSC lines (MUNIi008‐A, MUNIi009‐A, and MUNIi010‐A) derived and characterized in our laboratory [14]. Organoids were generated using a previously published protocol [17] with minor modifications as described in the Section 2. As shown in Fig. 1, all used iPSC lines can form mature cerebral organoids with typical morphology during development (Fig. 1A) and tissue patterning at day 55 (D55; Fig. 1B). Immunohistochemistry further confirmed that they expressed a series of progenitor as well as immature and mature neuronal markers, including PAX6, DCX, TUJ, MAP2, BRN2, and SYN1 (Fig. 1C). Thus, our data confirmed that COs at D55 express features of typical mature tissue and are suitable for our GLICO model experiments.

As a second component for the GLICO model, we used the U87, a well‐established glioblastoma cell line commonly used in brain cancer research [31]. To facilitate visualization of U87 within CO tissue, we fluorescently labeled this cell line using GFP or tdTomato constructs as described in Section 2. Under standard culture conditions, cells were maintained as an adherent culture and showed typical U87 morphology (Fig. 1D). Western blotting characterization demonstrated that the U87 cell line expressed a C‐MYC transcription factor. However, despite being of human brain origin, U87 did not express most of the analyzed neural or glial markers (i.e., SOX2, SOX1, NR2F2, Neurofilament Light chain (NF‐L), BRN2, N‐MYC, and GFAP), and showed very low or undetectable expression of neural stem marker PAX6 when compared to the self‐renewing and differentiating neural stem cell (NSC) line (ESI‐017 CoMoNSCs; [32]) (Fig. 1E). For setting up the GLICO model, glioblastoma cells were induced to form spheroids by transferring U87 cells to non‐adherent conditions. For each spheroid, 2000 cells were used. After 24 h, spheroids exhibited typical morphology with disorganized patterning (Fig. 1F) and were, at this time point, used for all our experiments.

### 3.2 Mature cerebral organoids support the growth of glioblastoma cells but need prolonged time for migration

**Fig. 2:**
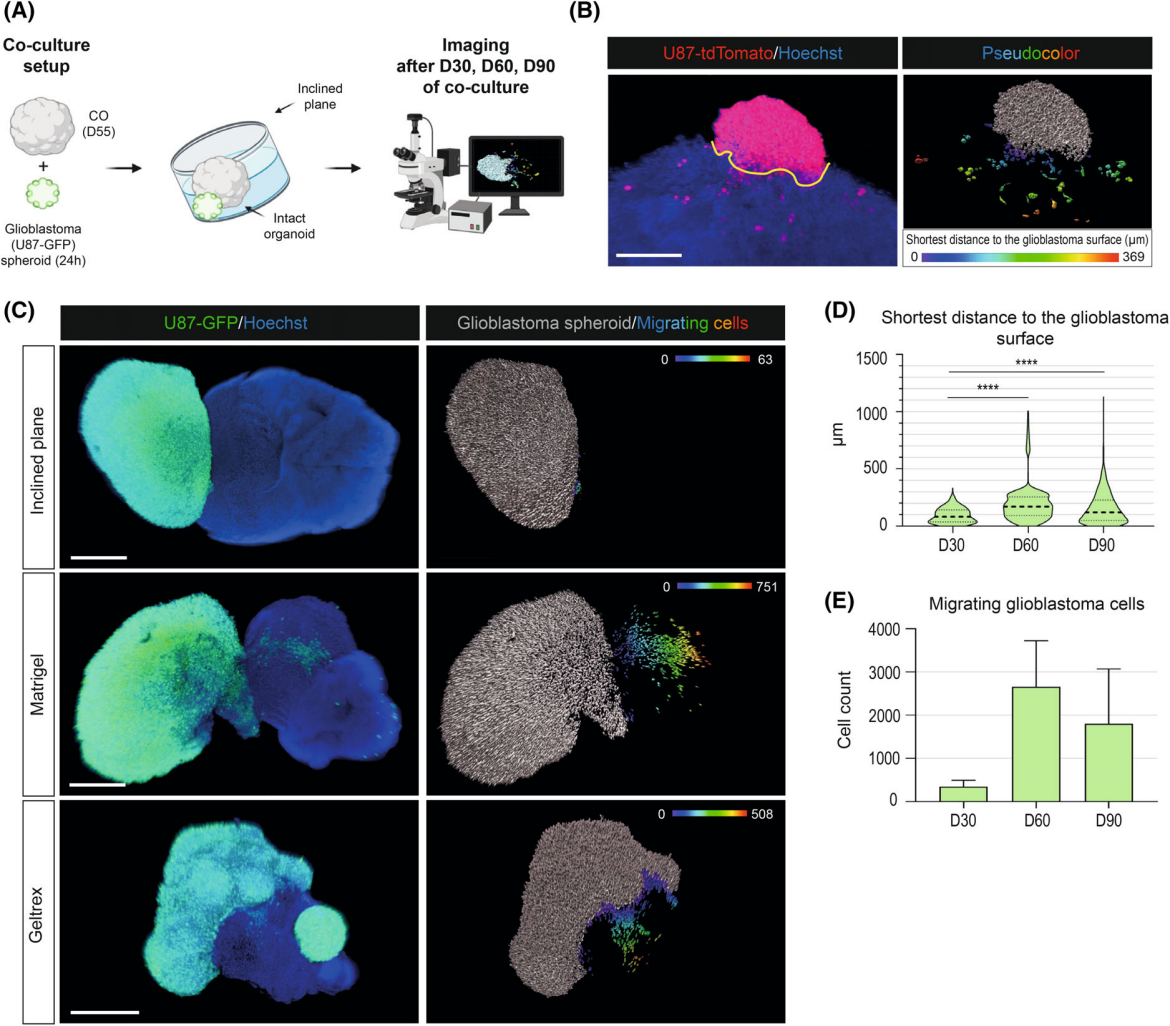
Mature cerebral organoids support the growth of glioblastoma cells but need prolonged time for migration. (A) A scheme showing the pipeline of glioblastoma‐cerebral organoid (GLICO) coculture using an inclined plane strategy. Twenty‐four‐hours‐old glioblastoma spheroid (U87) was transferred to the vicinity of a single 55‐day‐old mature cerebral organoid (CO) in a 12 well plate. The culture plate was tilted for 48 h to ensure the attachment of the glioblastoma spheroid to CO. Subsequently, the culture plate with GLICOs (COs attached to the glioblastoma spheroids) was transferred to the orbital shaker and maintained under standard CO cell culture conditions. Image acquisition and analysis were performed after 30, 60, and 90 days (D30, D60, and D90) of GLICO coculture. (B) Left image: cleared whole‐mount GLICO tissue depicting fluorescently labeled glioblastoma spheroid (red) and glioblastoma/CO nuclei (blue). The border between glioblastoma spheroid and migrating glioblastoma cells was determined manually using imaris software (schematically shown here as the yellow line). Scale bar = 300 μm. Right image: detection of individual glioblastoma cells using semi‐automatic image analysis methodology. Glioblastoma spheroid (gray) and cells migrated within CO tissue are marked using pseudocolor. A spectrum of colors corresponds to the shortest distance of individual cells to the delineated glioblastoma spheroid/CO border (μm). (C) Images of cleared whole‐mount GLICO tissues (left) indicate fluorescently labeled glioblastoma spheroid (green) and glioblastoma/CO nuclei (blue) at days 30, 60, and 90 of GLICO coculture. Visualization of individual glioblastoma cells (right) showing glioblastoma spheroid (gray) and cells migrated from the glioblastoma spheroid (pseudocolor). A spectrum of colors corresponds to the shortest distance of individual cells to the delineated glioblastoma spheroid/CO border (μm). Scale bar = 500 μm. Image analyses were performed from three independent coculture experiments with three GLICOs analyzed per each condition. (D) Violin plot showing the mean shortest migration distance (μm) of glioblastoma migrating cells to glioblastoma spheroid surface. Median of distance at D30 = 82.4 μm, Q1 = 37 μm, Q3 = 141 μm, median of distance at D60 = 170 μm, Q1 = 93.2 μm, Q3 = 256 μm, median of distance at D90 = 121 μm, Q1 = 49.8 μm, Q3 = 229 μm. ****P < 0.0001 by unpaired *t*‐test. (E) Bar graph showing the total count of cells migrating from the glioblastoma spheroid. Mean cell count at D30 = 340 migrating cells, mean cell count at D60 = 2659 migrating cells, mean cell count at D90 = 1797 migrating cells. Error bars represent the standard error of the mean. The analysis in (D) and (E) compares the length of cocultivation on days 30, 60, and 90 using an inclined plane. Data were collected from three independent experiments.

Having the primary components of the GLICO model characterized, we subsequently aimed to optimize (a) the coculture conditions that would mimic the *in vivo* conditions of glioblastoma growth as closely as possible and (b) the visualization of the glioblastoma and COs interactions. For the coculture, we first transferred a single 24‐h‐old glioblastoma spheroid to the vicinity of a single 55‐day‐old mature CO in a 12‐well plate. As depicted in Fig. 2A, we tilted the cell culture plate for 48 h to ensure the attachment of the glioblastoma spheroid and CO (we refer to this strategy as the ‘inclined plane’). Subsequently, COs with attached glioblastoma spheroids were transferred back to the orbital shaker and maintained under standard CO cell culture conditions. Samples for subsequent image analysis were harvested after 30, 60, and 90 days of GLICO coculture.

For visualization and image analysis of the GLICO model, we first used standard thin histological sections from paraffin embedding (~ 2 μm) or cryosections (~ 10 μm). However, this technique proved to be suboptimal as it did not allow us to track the complete attachment of glioblastoma spheroids onto COs and quantify the cell migration (data not shown). We thus optimized the protocol for the whole‐mount staining of CO tissue based on the published CUBIC protocol [21]. As shown in Fig. 2B, this approach (described in detail in Section 2) allowed us to image the whole GLICO tissue, specifically visualize all migrating cells within the CO and precisely quantify this migration. To do this, we first identified and delineated the border between the fluorescently labeled glioblastoma spheroid and healthy CO tissue (Hoechst‐positive cells; Fig. 2B – left panel). Subsequently, we visualized only fluorescently labeled cells and marked migrating cells within CO tissue using pseudocolors with a spectrum of colors corresponding to the distance from the delineated glioblastoma spheroid/CO border on the surface (Fig. 2B – right panel). In addition to visualization, we also quantified two parameters of cell migration, that is, (a) the shortest migratory distance from the glioblastoma spheroid/CO border and (b) the total number of migrating cells within one organoid (see below).

Having these parameters of visualization optimized, we evaluated the samples from three independent biological replicates harvested after 30, 60, and 90 days of GLICO inclined plane coculture. As shown in Fig. 2C, the extent of migration gradually increased over time in cell culture, with relatively minor migration observed at D30 that significantly increased at D60 and D90. Upon quantification, the data pointed out that although the longest distance of individual migrating glioblastoma cells to the surface of the glioblastoma sphere increased with time, the mean distance and the distance distribution were significantly higher at D60 (median of distance = 170 μm, Q1 = 93.2 μm, Q3 = 256 μm) and at D90 (median of distance = 121 μm, Q1 = 49.8 μm, Q3 = 229 μm) than at the D30 timepoint (median of distance = 82.4 μm, Q1 = 37 μm, Q3 = 141 μm; Fig. 2D). Additionally, as shown in Fig. 2E, the total number of migrating cells was very low at D30 (mean value = 340 migrating cells) and markedly increased at later time points (mean value at D60 = 2659 migrating cells; mean value at D90 = 1797 migrating cells), with D60 showing a trend toward a higher number of migrating glioblastoma cells detected within COs. Altogether, our data showed that coculture of COs with glioblastoma spheroids within 30–60 days represents the optimal setup for future analyses.

### 3.3 Coculture with ECM proteins enhances the growth and migration of glioblastoma

**Fig. 3:**
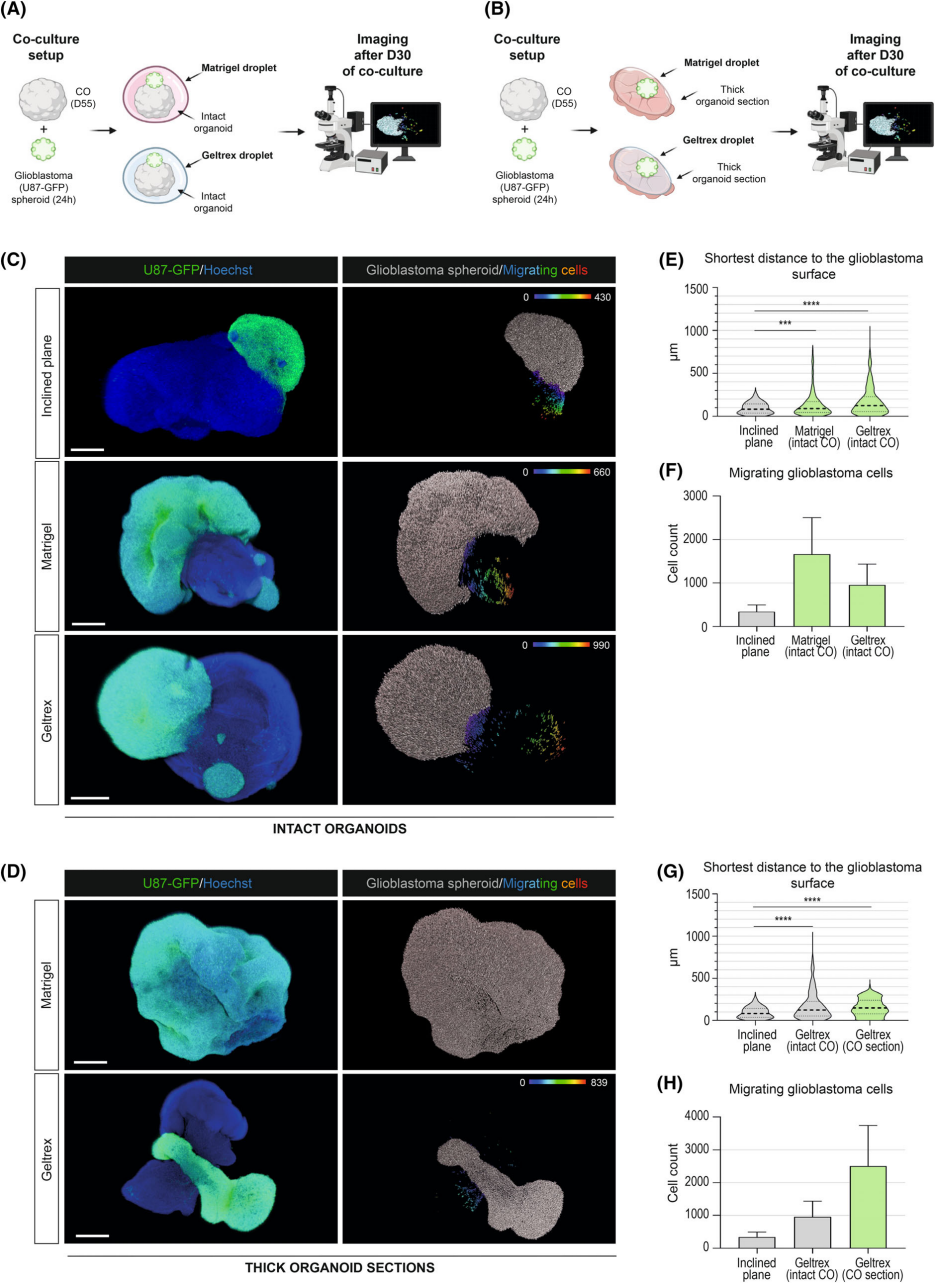
Coculture with ECM proteins enhances the growth and migration of glioblastoma. A scheme showing the pipeline of glioblastoma‐cerebral organoid (GLICO) coculture using intact 55‐day‐old organoids (A) or thick (250 μm) sections of cerebral organoids (COs) (B). 24‐h‐old glioblastoma spheroid (U87) was transferred to the vicinity of a single 55‐day‐old mature or thick (250 μm) section of COs. GLICO coculture was supported by embedding in a droplet of Matrigel™ or Geltrex™ at 37 °C for 10 min. GLICO was transferred into nonadherent cell culture dishes and cultured on an orbital shaker under standard CO cell culture conditions. Image acquisition and analysis were performed after 30 days of GLICO coculture. Images of cleared whole‐mount GLICO tissues after 30 days of coculture based on intact CO (C) or thick (250 μm) sections of COs (D) using Matrigel™ or Geltrex™. The left images show a fluorescently labeled glioblastoma spheroid (green) and glioblastoma/CO nuclei (blue). The right images visualize individual glioblastoma cells – glioblastoma spheroid (gray) and cells migrated from the glioblastoma spheroid (pseudocolor). In the case of Matrigel on organoid sections depicted in (F), the growth of glioblastoma increased to such an extent that it was impossible to evaluate the cell migration after 30 days. A spectrum of colors corresponds to the shortest distance of individual migrating cells to the delineated glioblastoma spheroid/CO border (μm). Scale bar = 500 μm. Three independent coculture experiments were used for the analysis with three GLICOs analyzed per each condition. (E, G) Violin plots showing the mean shortest migration distance (μm) of glioblastoma migrating cells to glioblastoma spheroid surface. Median distance with Matrigel in intact organoid = 89 μm, Q1 = 42.7 μm, Q3 = 171 μm; median distance with Geltrex in intact organoid = 123 μm, Q1 = 52.8 μm, Q3 = 227 μm; median distance with Geltrex in a thick section = 148 μm, Q1 = 78 μm, Q3 = 239 μm. ***P < 0.0005, ****P < 0.0001 by unpaired *t*‐test. (F, H) Bar graphs showing the total count of cells migrating from the glioblastoma spheroid. Mean cell count with Matrigel in intact organoid = 1661 migrating cells, Mean cell count with Geltrex in intact organoid = 954 migrating cells, Mean cell count with Geltrex in a thick section = 2505 migrating cells. Error bars represent the standard error of the mean. The analysis in (D, E) and (G, H) compare the use of Matrigel™ and Geltrex™ against an inclined plane on day 30 (shown in Fig. 2). Data were collected from three independent experiments.

Thus far, our optimized coculture approach indicated that a significantly longer time is needed to establish the GLICO model compared to some of the previously reported data (ranging from 2 days to 4 months; [6, 8, 9, 10, 11]). However, these reports used relatively young COs containing Matrigel as a scaffold or Matrigel to support GLICO coculture. As our mature COs (D55) were already devoid of ECM droplets, we aimed to analyze if these ECM protein scaffolds influence the glioblastoma cell migration within our GLICO model. As depicted in Fig. 3, we used two different approaches to test this hypothesis utilizing either (a) intact D55‐old organoids (Fig. 3A; as described above) or (b) thick (250 μm) sections of COs (Fig. 3B; as described in Section 2), mimicking organotypic brain slice cultures [33]. For both approaches, the coculture was supported with two types of commercially available ECM solutions: growth factors containing Matrigel or growth factor‐free Geltrex. All experimental conditions were fixed 30 days after the initiation of GLICO coculture, and the extent of migration was visualized and subsequently quantified as described above. Results from the intact coculture approach showed that both Matrigel and Geltrex significantly enhanced the migration of glioblastoma cells inside the COs (Fig. 3C) in comparison to the ECM‐free coculture system (Fig. 2C: Day 30). Quantification of the migration confirmed the increased distance of migrating cells from the glioblastoma/CO border (median distance with Matrigel = 89 μm, Q1 = 42.7 μm, Q3 = 171 μm, median distance with Geltrex = 123 μm, Q1 = 52.8 μm, Q3 = 227 μm; Fig. 3E) and increased the number of migrating cells (Matrigel mean value = 1661 migrating cells, Geltrex mean value = 954 migrating cells; Fig. 3F). In comparison to Geltrex, the presence of Matrigel had a more substantial effect on the number of migrating glioblastoma cells than on their distance from the glioblastoma/CO border.

Our second culture system using thick organoid sections confirmed the previous observations. As shown in Fig. 3D, the presence of Matrigel on organoid sections increased the growth of glioblastoma to such an extent that it was impossible to evaluate the cell migration after 30 days as the whole thick organoid section was overgrown by the glioblastoma. In the case of Geltrex, its presence also substantially stimulated the glioblastoma cell migration, but the parameters of cell migration from three independent biological replicates could be evaluated. Compared to intact organoids, thick CO sections showed an increased median distance of migration (148 μm, Q1 = 78 μm, Q3 = 239 μm) and the total number of migrating glioblastoma cells (mean value = 2505 migrating cells) within the CO tissue (Fig. 3G,H). Notably, migration parameters within organoid sections supported with Geltrex at D30 were comparable to that of the inclined plane at D60. Our results thus confirm that both used ECM protein scaffolds significantly enhance the glioblastoma cell growth and migration, with the growth factors containing Matrigel being more potent. It is of note that while both Matrigel and Geltrex contain ECM proteins that support the proliferation and growth of glioblastoma cells, the composition of both products differs from ECM proteins found in the human brain. Brain‐specific ECM contains mostly proteoglycans and glycosaminoglycans and low levels of fibrous proteins such as collagen and fibronectin [34, 35, 36]. On the contrary, Geltrex and Matrigel are mainly composed of laminin and collagen and are of mouse origin [37]. We thus believe the inclined plane method reflects more closely the situation found in humans *in vivo*. Importantly, to what extent these protein cocktails influence the biological properties of glioblastoma cultured within the GLICO model remains to be investigated.

### 3.4 Coculture of glioblastoma cells with COs significantly changes their cell identity toward proneural cell type

**Fig. 4:**
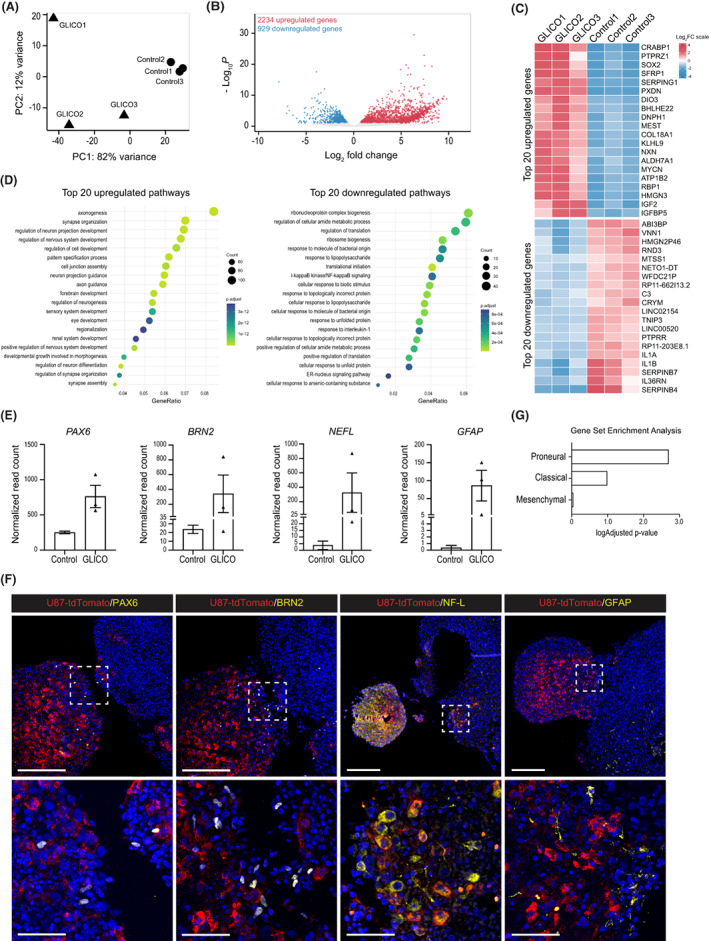
Coculture of glioblastoma cells with COs significantly changes their cell identity toward proneural cell type. (A) Principal component analysis (PCA) showing the differences in gene expression between three independent control U87 spheroid samples (Control 1, Control 2, Control 3) and three independent samples of U87 spheroids cocultured within the glioblastoma‐cerebral organoid (GLICO) model for 40 days (GLICO1, GLICO2, GLICO3). Five GLICOs were pooled in each replicate. (B) Volcano plot showing significantly upregulated (red) and downregulated (blue) differentially expressed genes (DEGs) in U87 cocultured within the GLICO model vs. control U87 spheroids. (C) Heatmap showing 20 most upregulated and 20 most downregulated DEGs in three samples of U87 cocultured within the GLICO model (GLICO1, GLICO2, GLICO3) and three control U87 spheroids samples (Control 1, Control 2, Control 3). (D) Over‐representation analysis (ORA) showing 20 most upregulated (left) and 20 most downregulated (right) pathways in U87 cocultured within the GLICO model categorized based on gene ontology analysis. (E) Normalized read counts of selected markers (*PAX6*, *BRN2*, *NEFL*, *and GFAP*) from bulk mRNA sequencing data of control U87 spheroids (Control) and U87 cocultured within the GLICO model (GLICO). (F) Representative IHC staining images of U87 cocultured within the GLICO model showing the localization of selected markers PAX6, BRN2, NF‐L, and GFAP (yellow) in the U87 spheroid labeled with tdTomato (red). Scale bar = 200 μm. The bottom images show magnification of the area marked with a white square in the respective top images. Scale bar = 50 μm. (G) Enrichment of a custom list of genes in GLICO samples specific for different glioblastoma subtypes defined in [30, 38]. Data were collected from three independent experiments.

Finally, to evaluate our GLICO model from the molecular perspective, we used bulk mRNA sequencing as a model experiment. Using this approach, we determined changes in the gene expression of GFP^+^ glioblastoma cells cultured as spheroids for 40 days (Control) or cocultured with COs within the GLICO model for 40 days without any ECM proteins. As shown in Fig. 4A, principal component analysis (PCA) revealed substantial differences induced in the U87 glioblastoma cells upon 40 days of coculture with the GLICO model. While three independent replicates of U87 glioblastoma cell line cultured as spheroids clustered together, three independent replicates of U87 cocultured with COs (*n* = 5 GLICOs in each replicate) were separated from glioblastoma spheroids and showed broader distribution across the PCA plot. Visualization of significantly upregulated and downregulated genes is shown in the volcano plot (Fig. 4B), with the top 20 differentially expressed genes (DEGs) in each category depicted in Fig. 4C. Interestingly, over‐representation analysis (ORA) revealed that genes most significantly upregulated in glioblastoma cells upon co‐culture with COs were related to the regulation of neural development, neurogenesis, brain development, and morphogenesis (Fig. 4D). On the contrary, significantly downregulated genes were most prominently related to cytokine signaling pathway and production, regulation of secretion, and inflammatory response (Fig. 4D). This data indicated that U87 glioblastoma cells substantially changed their expression profile toward neural cell fate.

To validate the data from bulk mRNA seq, we selected a set of neural and glial‐specific genes (i.e., PAX6, BRN2/*POU3F2*, NF‐L/*NEFL*, and GFAP) showing high expression in the GLICO model in comparison to independently cultured glioblastoma spheroids (Fig. 4E) or standard glioblastoma 2D culture (Fig. 1E). We then performed their immunohistological staining in glioblastoma cocultured with COs and confirmed that, unlike the original U87 cell line (Fig. 1E), U87 cells cocultured with COs for 30 days specifically express markers of neuronal and glial cell fate (Fig. 4F). To also analyze the dynamics of the expression of neural markers in U87 glioblastoma cells during coculture with COs, we performed a qPCR analysis of the selected neural markers on D20 and D40 of GLICO coculture. Our data show that the expression of *GFAP*, *NEFL*, and *SOX2* gradually increases in U87 cells from the beginning of coculture with COs (Fig. S1). However, the expression of *PAX6* and *MAP2* was induced only later during the GLICO co‐culture, underlying the different dynamics of the activation of specific markers (Fig. S1).

Lastly, prompted by these observations, we compared our dataset to a gene expression database of clinically relevant glioblastoma subtypes [30, 38]. This comparison revealed that upon coculture of U87 with COs, glioblastoma cells significantly changed their identity from mesenchymal to proneural and, to some extent, also to classical glioblastoma cell type (Fig. 4G and Fig. S2). Thus, taken together, our data point out that the CO‐specific microenvironment has the potential to significantly alter the identity of cocultured glioblastoma and change its biological properties.

## 4 Discussion

In this study, we show that mature, 55‐day‐old COs derived from three independent iPSC lines support the growth and migration of the glioblastoma model cell line U87. Furthermore, we point out that the use of ECM proteins (i.e., Matrigel and Geltrex) significantly enhances the migration of U87 cells inside the intact or sectioned organoids. We also describe methods for clear and precise visualization and quantification of migrating glioblastoma cells within intact mature organoids and quantify the extent of migration in each of the culture conditions used. Finally, we bring evidence that this coculture model transforms the identity of an established glioblastoma cell line U87 toward proneural and classical glioblastoma cell types, suggesting that the CO‐specific microenvironment has the potential to ‘reprogram’ the long‐established U87 glioblastoma cell line.

To this date, seven reports have provided evidence that glioblastoma coculture with COs could be a way to overcome the challenges of inadequate 2D cell cultures and demanding *in vivo* PDX models. These reports have used GLICO models (a) to demonstrate the possibility to coculture COs with glioblastoma [6, 8]; (b) to show that GLICO tumor biology is similar to that found in patients or animal models [9, 10, 11]; or (c) to provide an insight into molecular players behind the biology of glioblastoma and its microenvironment using a single‐cell sequencing approach [7, 10, 12]. However, these studies markedly differed in numerous technical aspects of GLICO coculture (such as the use of COs derived from mice vs. human iPSCs, the use of 12 day old COs to 4‐month‐old COs, and the use of Matrigel, to name a few). These differences prompted us to explore the optimal conditions for the GLICO models and which technical parameters are crucial for the invasiveness of glioblastoma. Our results showed that mature, 55‐day‐old human COs from three individual iPSC lines could support the growth of U87 glioblastoma cells upon simple coculture on an inclined plane.

However, we discovered that it took at least 30 days to detect migrating U87 glioblastoma cells in COs, with the majority of migrating cells being detectable around D60 of GLICO coculture. This observation was in strong contrast to previous reports showing that glioblastoma migration began within several days from the initiation of coculture [6, 7, 8, 9, 10, 11, 12]. Since our result was observed repeatedly and independently verified using three different iPSC lines with the same outcome, there are several possible explanations for this finding, including (a) the specific properties of the glioblastoma cell line, (b) the age of COs used, or (c) other parameters of co‐culture conditions. Indeed, it has been demonstrated that significant differences in migration are caused by the primary GSCs or glioblastoma cell lines used [6, 9]. Ogawa et al. [6] showed that the invasiveness of patient‐derived glioblastoma cell lines in COs correlated with lethality in mice and Goranci‐Buzhala et al. [9] also noted different migratory capacities between primary and recurrent patient‐derived glioblastoma lines. Thus the significantly longer time needed for the migration of U87 could be partially caused by the nature of our experimental cell line.

Additionally, it is possible that the age of COs may influence the migration of grafted cells. Previously, Goranci‐Buzhala et al. [9] compared 20‐, 40‐, and 60 day old COs and found that increasing maturity correlated with the increased integration of glioblastoma. And while Linkous et al. [11] did not see changes in glioblastoma growth rates in 1‐ and 4 month old organoids, they did observe a difference in the growth patterns. GLICO tumors from younger, 1‐month‐old COs exhibited large areas of regional proliferation, whereas GLICO tumors from older, 4‐month‐old COs displayed a more infiltrative growth pattern. This suggests that cytoarchitecture or specific components of the mature organoid microenvironment can influence the migration and spread of the tumor.

Lastly, we wanted to verify how other parameter(s) of coculture, such as the use of ECM proteins, could influence the ability of glioblastoma cells to migrate within COs. Indeed, upon detailed inspection of every GLICO report published to this date, we found that reports most often used young COs (D12–D30) that are usually (at this early time point) embedded in Matrigel for proper maturation. If older COs were used (that are usually devoid of Matrigel embedding), then Matrigel was utilized as a scaffold to support GLICO coculture or added directly to the organoid growth medium. This prompted us to test whether ECM proteins could cause the observed discrepancy and enhance the glioblastoma cell growth. We thus examined two commercially available products: commonly used Matrigel, containing a mixture of growth factors, and Geltrex, a growth factor‐free alternative. Our results demonstrated that both tested ECM protein mixtures markedly increased the number of migrating cells, but not the migratory distance, compared to simple GLICO coculture without ECM proteins. This effect was significantly enhanced when thick organoid sections were used for coculture instead of intact organoids. Under this condition, coculture with Matrigel for 30 days resulted in complete overgrowth of the CO section, implying that a much shorter time could be used when analyzing the migrating cells within this model. The coculture with Geltrex for 30 days also enhanced the glioblastoma growth, but it was comparable to a 60‐day‐long coculture on an inclined plane. These results thus show that ECM proteins markedly enhance the glioblastoma growth within the COs, and thus, any data on cell invasiveness and migration should be interpreted with caution. Additionally, it would be compelling to analyze to what extent these protein cocktails influence the biological properties of glioblastoma cultured within the GLICO model. This, however, remains to be investigated in the future.

Notably, while setting up the GLICO coculture model to subsequently evaluate and quantify the migratory glioblastoma cells, we found out that such quantification would not be possible using standard histological sections. We thus optimized the tissue clearing method previously used for the whole‐brain and whole‐body clearing [21]. This method is similar to that implemented recently by [9] for the GLICO model. Additionally, we also developed a semi‐automated pipeline to visualize and quantify migrating cells using commercially available software imaris. This pipeline, which we describe in detail in Section 2, allows users to quantitatively evaluate the migration under different cell culture conditions (as described above).

Finally, we aimed to demonstrate that it was possible to isolate specifically the fluorescently labeled glioblastoma cells after a long coculture period with COs and analyze them separately from the bulk tissue. We, thus, performed a proof‐of‐concept experiment and used GFP^+^ sorted glioblastoma cells and their respective controls for bulk mRNA seq. Notably, previous reports have shown that glioblastomas cocultured with COs retain a similar gene expression profile to the original tumor tissue, proving that the coculture system is superior to standard 2D culture, possibly due to the effect of a healthy brain microenvironment [12]. Pine et al. also showed that this coculture enhanced the expression of proneural‐ and to some extent also, classical‐type glioblastoma‐specific genes. Our results confirm this finding and demonstrate that the CO microenvironment can stimulate significant changes in gene expression even in the well‐established glioblastoma cell line U87 previously cultured in 2D cell culture conditions with FBS in the culture medium. We show that the newly acquired gene expression profile is closely related to neuronal development, neurogenesis maturation, and axon guidance, suggesting that U87 glioblastoma acquired neural identity upon coculture. Some of these results were verified using immunohistochemistry, thus proving that observed results from bulk mRNA correspond to the newly acquired U87 glioblastoma phenotype. Importantly, a comparison to a clinically relevant gene dataset for glioblastoma subtypes [30] confirmed also in our dataset that GLICO coculture changed the identity toward proneural and classical cell types. This is a remarkable finding since it not only supports the findings of Pine et al. [12] but mainly demonstrates that the CO microenvironment has the potential to ‘reprogram’ the established gene expression of such a cell line as U87.

Lastly, our study has several limitations which remain to be addressed in the future. Major limitation is the use of U87 cell line, which, despite being a bona fide human glioblastoma cell line, does not adequately represent the patient‐derived tumor [39]. Thus, more experimental repeats with several cell lines would strengthen the provided proof‐of‐concept data. Additionally, detailed analysis of how organoid cytoarchitecture affects the invasion routes of glioblastoma has not been performed. Which specific organoid compartments influence the glioblastoma migratory phenotype thus remains to be explored.

## 5 Conclusions

Taken together, our results demonstrate that mature, 55‐day‐old cerebral organoids support the growth of glioblastoma cells *in vitro*, and their integration and migration into organoids can be significantly enhanced using ECM protein scaffolds, such as Matrigel or Geltrex. We also describe a pipeline for visualization and quantification of migrating cells within the whole organoid tissue. Lastly, our proof‐of‐concept bulk mRNA seq analysis brings evidence that the coculture of the U87 cell line with a human brain‐like microenvironment significantly changes the glioblastoma cell identity. And while it has been previously reported that the GLICO model faithfully preserves the characteristics of the patient‐resected tissue in the *in vitro* culture, we, for the first time, demonstrate that this model is also capable of transforming the gene expression profile of established U87 glioblastoma cell line into more *in vivo*‐related phenotype. Which elements of the brain‐like microenvironment within cerebral organoids are driving these changes remains to be investigated.

## Conflict of interest

The authors declare no conflict of interest.

## Author contributions

VF, VP, TV, KAC, ZH, and DB: designed the project and wrote the manuscript with input from all authors; VF, VP, TV, KAC, JS, JR, SV, and JH: performed experiments, collected and analyzed the data; PA, ZM, and LV: performed bioinformatic analysis and visualizations; DB, ZH, and LV: interpreted data, revised the manuscript.

### Peer review

The peer review history for this article is available at https://publons.com/publon/10.1002/1878‐0261.13389.

## Supporting information


**Fig. S1.** The onset of the expression of neural markers in glioblastoma cells during coculture within the GLICO model.

**Fig. S2.** A comparison of our dataset to a gene expression database of clinically relevant glioblastoma subtypes.

**Table S1.** List of antibodies used for western blotting.
**Table S2.** List of antibodies used for immunohistochemistry.
**Table S3.** Whole‐mount and CUBIC clearing buffer compositions.
**Table S4.** List of primer sets used for qPCR.

## Data Availability

The raw research data are available per request through the corresponding author. Final data from mRNA seq analyses were deposited under GEO accession number GSE216626.

## References

[1] Ostrom QT , Gittleman H , Liao P , Rouse C , Chen Y , Dowling J , et al. CBTRUS statistical report: primary brain and central nervous system tumors diagnosed in the United States in 2007–2011. Neuro Oncol. 2014;16(Suppl 4):iv1–63. 25304271 10.1093/neuonc/nou223 PMC4193675

[2] Stupp R , Hegi ME , Mason WP , van den Bent M , Taphoorn MJ , Janzer RC , et al. Effects of radiotherapy with concomitant and adjuvant temozolomide versus radiotherapy alone on survival in glioblastoma in a randomised phase III study: 5‐year analysis of the EORTC‐NCIC trial. Lancet Oncol. 2009;10:459–66. 19269895 10.1016/S1470-2045(09)70025-7

[3] Poon MTC , Sudlow CLM , Figueroa JD , Brennan PM . Longer‐term (≥ 2 years) survival in patients with glioblastoma in population‐based studies pre‐ and post‐2005: a systematic review and meta‐analysis. Sci Rep. 2020;10:11622. 32669604 10.1038/s41598-020-68011-4 PMC7363854

[4] Lancaster MA , Renner M , Martin C‐A , Wenzel D , Bicknell LS , Hurles ME , et al. Cerebral organoids model human brain development and microcephaly. Nature. 2013;501:373–9. 23995685 10.1038/nature12517 PMC3817409

[5] Bian S , Repic M , Guo Z , Kavirayani A , Burkard T , Bagley JA , et al. Genetically engineered cerebral organoids model brain tumour formation. Nat Methods. 2018;15:631–9. 30038414 10.1038/s41592-018-0070-7 PMC6071863

[6] Ogawa J , Pao GM , Shokhirev MN , Verma IM . Glioblastoma model using human cerebral organoids. Cell Rep. 2018;23:1220–9. 29694897 10.1016/j.celrep.2018.03.105 PMC6892608

[7] Bhaduri A , Di Lullo E , Jung D , Müller S , Crouch EE , Espinosa CS , et al. Outer radial glia‐like cancer stem cells contribute to heterogeneity of glioblastoma. Cell Stem Cell. 2020;26:48–63.e6. 31901251 10.1016/j.stem.2019.11.015 PMC7029801

[8] da Silva B , Mathew RK , Polson ES , Williams J , Wurdak H . Spontaneous glioblastoma spheroid infiltration of early‐stage cerebral organoids models brain tumor invasion. SLAS Discov. 2018;23:862–8. 29543559 10.1177/2472555218764623

[9] Goranci‐Buzhala G , Mariappan A , Gabriel E , Ramani A , Ricci‐Vitiani L , Buccarelli M , et al. Rapid and efficient invasion assay of glioblastoma in human brain organoids. Cell Rep. 2020;31:107738. 32521263 10.1016/j.celrep.2020.107738

[10] Krieger TG , Tirier SM , Park J , Jechow K , Eisemann T , Peterziel H , et al. Modeling glioblastoma invasion using human brain organoids and single‐cell transcriptomics. Neuro Oncol. 2020;22:1138–49. 32297954 10.1093/neuonc/noaa091 PMC7594554

[11] Linkous A , Balamatsias D , Snuderl M , Edwards L , Miyaguchi K , Milner T , et al. Modeling patient‐derived glioblastoma with cerebral organoids. Cell Rep. 2019;26:3203–11.e5. 30893594 10.1016/j.celrep.2019.02.063 PMC6625753

[12] Pine AR , Cirigliano SM , Nicholson JG , Hu Y , Linkous A , Miyaguchi K , et al. Tumor microenvironment is critical for the maintenance of cellular states found in primary glioblastomas. Cancer Discov. 2020;10:964–79. 32253265 10.1158/2159-8290.CD-20-0057 PMC10256258

[13] Mariappan A , Goranci‐Buzhala G , Ricci‐Vitiani L , Pallini R , Gopalakrishnan J . Trends and challenges in modeling glioma using 3D human brain organoids. Cell Death Differ. 2021;28:15–23. 33262470 10.1038/s41418-020-00679-7 PMC7707134

[14] Raska J , Hribkova H , Klimova H , Fedorova V , Barak M , Barta T , et al. Generation of six human iPSC lines from patients with a familial Alzheimer's disease (n = 3) and sex‐ and age‐matched healthy controls (n = 3). Stem Cell Res. 2021;53:102379. 34088008 10.1016/j.scr.2021.102379

[15] Raska J , Klimova H , Sheardova K , Fedorova V , Hribkova H , Pospisilova V , et al. Generation of three human iPSC lines from patients with a spontaneous late‐onset Alzheimer's disease and three sex‐ and age‐matched healthy controls. Stem Cell Res. 2021;53:102378. 34088007 10.1016/j.scr.2021.102378

[16] Camp JG , Badsha F , Florio M , Kanton S , Gerber T , Wilsch‐Bräuninger M , et al. Human cerebral organoids recapitulate gene expression programs of fetal neocortex development. Proc Natl Acad Sci USA. 2015;112:15672–7. 26644564 10.1073/pnas.1520760112 PMC4697386

[17] Lancaster MA , Knoblich JA . Generation of cerebral organoids from human pluripotent stem cells. Nat Protoc. 2014;9:2329–40. 25188634 10.1038/nprot.2014.158 PMC4160653

[18] Besse A , Sana J , Lakomy R , Kren L , Fadrus P , Smrcka M , et al. MiR‐338‐5p sensitizes glioblastoma cells to radiation through regulation of genes involved in DNA damage response. Tumor Biol. 2016;37:7719–27. 10.1007/s13277-015-4654-x 26692101

[19] Ondracek J , Fadrus P , Sana J , Besse A , Loja T , Vecera M , et al. Global MicroRNA expression profiling identifies unique MicroRNA pattern of radioresistant glioblastoma cells. Anticancer Res. 2017;37:1099–104. 28314270 10.21873/anticanres.11422

[20] Fedorova V , Vanova T , Elrefae L , Pospisil J , Petrasova M , Kolajova V , et al. Differentiation of neural rosettes from human pluripotent stem cells in vitro is sequentially regulated on a molecular level and accomplished by the mechanism reminiscent of secondary neurulation. Stem Cell Res. 2019;40:101563. 31494448 10.1016/j.scr.2019.101563

[21] Susaki EA , Tainaka K , Perrin D , Yukinaga H , Kuno A , Ueda HR . Advanced CUBIC protocols for whole‐brain and whole‐body clearing and imaging. Nat Protoc. 2015;10:1709–27. 26448360 10.1038/nprot.2015.085

[22] Wingett SW , Andrews S . FastQ screen: a tool for multi‐genome mapping and quality control. F1000Res. 2018;7:1338. 30254741 10.12688/f1000research.15931.1 PMC6124377

[23] Dobin A , Davis CA , Schlesinger F , Drenkow J , Zaleski C , Jha S , et al. STAR: ultrafast universal RNA‐seq aligner. Bioinformatics. 2013;29:15–21. 23104886 10.1093/bioinformatics/bts635 PMC3530905

[24] Kopylova E , Noé L , Touzet H . SortMeRNA: fast and accurate filtering of ribosomal RNAs in metatranscriptomic data. Bioinformatics. 2012;28:3211–7. 23071270 10.1093/bioinformatics/bts611

[25] Love MI , Huber W , Anders S . Moderated estimation of fold change and dispersion for RNA‐seq data with DESeq2. Genome Biol. 2014;15:550. 25516281 10.1186/s13059-014-0550-8 PMC4302049

[26] org.Hs.eg.db . Bioconductor. Available at http://bioconductor.org/packages/org.Hs.eg.db/. Accessed 14 April 2022.

[27] Blighe K. EnhancedVolcano: publication‐ready volcano plots with enhanced colouring and labeling. 2022.

[28] Wu T , Hu E , Xu S , Chen M , Guo P , Dai Z , et al. clusterProfiler 4.0: a universal enrichment tool for interpreting omics data. Innovation. 2021;2:100141. 34557778 10.1016/j.xinn.2021.100141 PMC8454663

[29] Yu G , Wang L‐G , Han Y , He QY . clusterProfiler: an R package for comparing biological themes among gene clusters. OMICS. 2012;16:284–7. 22455463 10.1089/omi.2011.0118 PMC3339379

[30] Verhaak RGW , Hoadley KA , Purdom E , Wang V , Qi Y , Wilkerson MD , et al. Integrated genomic analysis identifies clinically relevant subtypes of glioblastoma characterized by abnormalities in PDGFRA, IDH1, EGFR, and NF1. Cancer Cell. 2010;17:98–110. 20129251 10.1016/j.ccr.2009.12.020 PMC2818769

[31] Clark MJ , Homer N , O'Connor BD , Chen Z , Eskin A , Lee H , et al. U87MG decoded: the genomic sequence of a cytogenetically aberrant human cancer cell line. PLoS Genet. 2010;6:e1000832. 20126413 10.1371/journal.pgen.1000832 PMC2813426

[32] Bohaciakova D , Hruska‐Plochan M , Tsunemoto R , Gifford WD , Driscoll SP , Glenn TD , et al. A scalable solution for isolating human multipotent clinical‐grade neural stem cells from ES precursors. Stem Cell Res Ther. 2019;10:83. 30867054 10.1186/s13287-019-1163-7 PMC6417180

[33] Marques‐Torrejon MA , Gangoso E , Pollard SM . Modelling glioblastoma tumour‐host cell interactions using adult brain organotypic slice co‐culture. Dis Model Mech. 2018;11:dmm031435. 29196443 10.1242/dmm.031435 PMC5894940

[34] Dityatev A , Schachner M , Sonderegger P . The dual role of the extracellular matrix in synaptic plasticity and homeostasis. Nat Rev Neurosci. 2010;11:735–46. 20944663 10.1038/nrn2898

[35] Song I , Dityatev A . Crosstalk between glia, extracellular matrix and neurons. Brain Res Bull. 2018;136:101–8. 28284900 10.1016/j.brainresbull.2017.03.003

[36] Lam D , Enright HA , Cadena J , Peters SKG , Sales AP , Osburn JJ , et al. Tissue‐specific extracellular matrix accelerates the formation of neural networks and communities in a neuron‐glia co‐culture on a multi‐electrode array. Sci Rep. 2019;9:4159. 30858401 10.1038/s41598-019-40128-1 PMC6411890

[37] Aisenbrey EA , Murphy WL . Synthetic alternatives to Matrigel. Nat Rev Mater. 2020;5:539–51. 32953138 10.1038/s41578-020-0199-8 PMC7500703

[38] Wang Q , Hu B , Hu X , Kim H , Squatrito M , Scarpace L , et al. Tumor evolution of glioma‐intrinsic gene expression subtypes associates with immunological changes in the microenvironment. Cancer Cell. 2017;32:42–56.e6. 28697342 10.1016/j.ccell.2017.06.003 PMC5599156

[39] Allen M , Bjerke M , Edlund H , Nelander S , Westermark B . Origin of the U87MG glioma cell line: good news and bad news. Sci Transl Med. 2016;8:354re3. 10.1126/scitranslmed.aaf6853 27582061

