# Supplementary figures and images for: Glioblastoma and cerebral organoids: development and analysis of an in vitro model for glioblastoma migration
Source: Mol Oncol. 2023 Feb 18;17(4):647–63. doi: 10.1002/1878-0261.13389 (PMC10061278; doi:10.1002/1878-0261.13389)

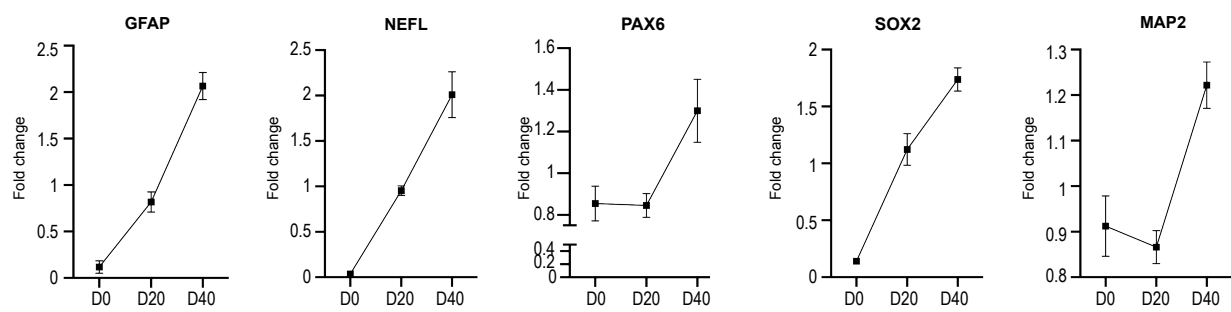

Supplementary Figure 1

Supplement: Supplementary file 1 — Fig. S1. The onset of the expression of neural markers in glioblastoma cells during coculture within the GLICO model. [file MOL2-17-647-s001.pdf]

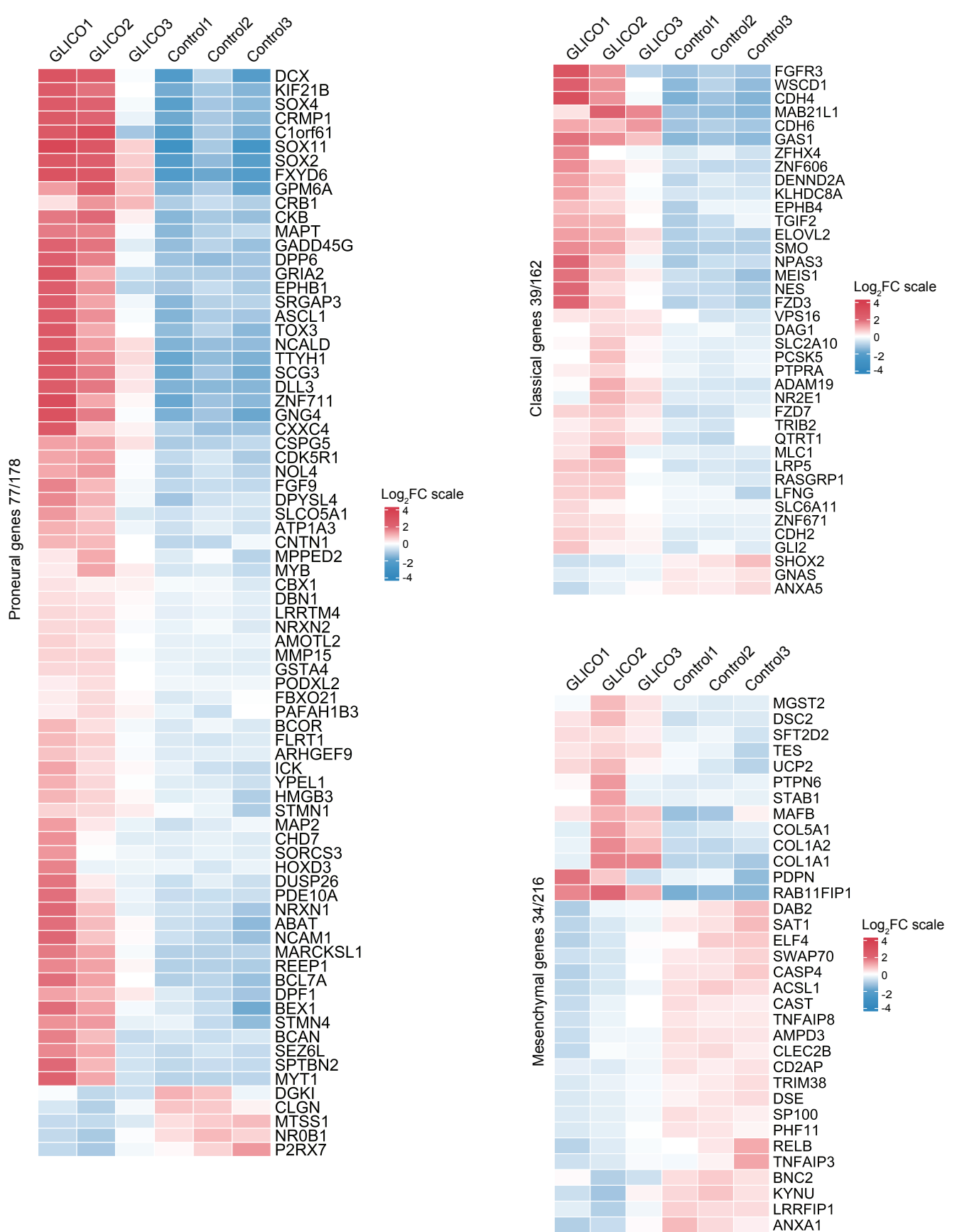

Supplementary Figure 2

Supplement: Supplementary file 2 — Fig. S2. A comparison of our dataset to a gene expression database of clinically relevant glioblastoma subtypes. [file MOL2-17-647-s002.pdf]
