# Supplementary material for: Glioblastoma and cerebral organoids: development and analysis of an in vitro model for glioblastoma migration
Source: Mol Oncol. 2023 Feb 18;17(4):647–63. doi: 10.1002/1878-0261.13389 (PMC10061278; doi:10.1002/1878-0261.13389)
Supplement: Supplementary file 3 — Table S1. List of antibodies used for western blotting. Table S2. List of antibodies used for immunohistochemistry. Table S3. Whole‐mount and CUBIC clearing buffer compositions. Table S4. List of primer sets used for qPCR. [file MOL2-17-647-s003.docx]

**Supplementary Material**

Figure S1. The onset of the expression of neural markers in glioblastoma cells during co-culture within the GLICO model

Figure S2. A comparison of our dataset to a gene expression database of clinically relevant glioblastoma subtypes

Supplementary Table 1: List of antibodies used for Western blotting

Supplementary Table 2: List of antibodies used for Immunohistochemistry

Supplementary Table 3: Whole-mount and CUBIC clearing buffer compositions

Supplementary Table 4: List of primer sets used for qPCR

**Figure S1. The onset of the expression of neural markers in glioblastoma cells during co-culture within the GLICO model**

qPCR analysis of selected neural markers (*GFAP, NEFL, PAX6, SOX2, MAP2*) in U87 cells after co-culture within the glioblastoma-–cerebral organoid (GLICO) model for 20 and 40 days. Day 0 (D0) represents control U87 spheroids before co-culturing with COs. Data were collected from one experiment (GFP+ isolated cells from 10-20 GLICOs were pooled for each sample). Error bars represent three technical replicates.

**Figure S2. A comparison of our dataset to a gene expression database of clinically relevant glioblastoma subtypes**

Heatmaps depicting genes from [30] with significantly changed expression in our datasets. Each heatmap represents one clinically relevant glioblastoma subtype (Proneural, Classical, and Mesenchymal; [38]).

**Supplementary Table 1: List of antibodies used for Western blotting**

| **Antibody** | **Manufacturer** | **Identifier** |
| --- | --- | --- |
| C-MYC (D84C12) | Cell Signaling Technology | Cat# 5605  RRID:AB_1903938 |
| SOX2 (L1D6A2) | Cell Signaling Technology | Cat# 4900  RRID: AB_10560516 |
| SOX1 | Cell Signaling Technology | Cat# 4194  RRID: AB_1904140 |
| PAX6 (D3A9V) | Cell Signaling Technology | Cat# 60433  RRID: AB_2797599 |
| NR2F2 (D16C4) | Cell Signaling Technology | Cat# 6434  RRID:AB_11220428 |
| NF-L (C28E10) | Cell Signaling Technology | Cat# 2837  RRID:AB_823575 |
| BRN2 (D2C1L) | Cell Signaling Technology | Cat# 12137  RRID:AB_2797827 |
| N-MYC (D1V2A) | Cell Signaling Technology | Cat# 84406  RRID:AB_2800038 |
| GFAP (D1F4Q) | Cell Signaling Technology | Cat# 12389  RRID:AB_2631098 |
| ß-ACTIN (8H10D10) | Cell Signaling Technology | Cat# 3700  RRID: AB_2242334 |

**Supplementary Table 2: List of antibodies used for Immunohistochemistry**

| **Antibody** | **Manufacturer** | **Identifier** |
| --- | --- | --- |
| BRN2 (D2C1L) | Cell Signaling Technology | Cat# 12137  RRID:AB_2797827 |
| DCX (E-6) | Santa Cruz Biotechnology | Cat# sc-271390;  RRID: X |
| GFAP (D1F4Q) | Cell Signaling Technology | Cat# 12389  RRID:AB_2631098 |
| MAP2 (D5G1) | Cell Signaling Technology | Cat# 8707;  RRID: AB_2722660 |
| NF-L (C28E10) | Cell Signaling Technology | Cat# 2837  RRID:AB_823575 |
| PAX6 (D3A9V) | Cell Signaling Technology | Cat# 60433  RRID: AB_2797599 |
| Synapsin-1 (D12G5) | Cell Signaling Technology | Cat# 5297;  RRID: AB_2616578 |
| TUJ (TU-20) | Cell Signaling Technology | Cat# 4466;  RRID: AB_1904176 |

**Supplementary Table 3: Whole-mount and CUBIC clearing buffer compositions**

| CUBIC1 | 25% urea (Merck), 25% N,N,N′,N′‑tetrakis(2‑hydroxypropyl)ethylene (Merck) and 15% Triton X-100 |
| --- | --- |
| Washing buffer | 0.5% Triton X-100 in PBS |
| Blocking buffer | PBS, 5% normal goat serum, 0.5% Triton X‑100, 0.01% NaN_3_ |
| CUBIC2 | 50% sucrose, 25% urea, 10% triethanolamine (Merck) and 0.1% Triton X-100 |
| Mounting solution | 1% agarose in CUBIC2 and PBS 3:1 |

**Supplementary Table 4: List of primer sets used for qPCR**

| **Gene name** | **Primer Sequence** | |
| --- | --- | --- |
| *GFAP* | F: CCGACAGCAGGTCCATGT | R: GTTGCTGGACGCCATTG |
| *MAP2* | F: TTGGTGCCGAGTGAGAAGA | R: GTCTGGCAGTGGTTGGTTAA |
| *NEFL* | F: CGACAGCTTGATGGACGAAAT | R: GATCTGCGCGTACTGGATCTG |
| *PAX6* | F: CTGAGGAATCAGAGAAGACAGGC | R: ATGGAGCCAGATGTGAAGGAGG |
| *SOX2* | F: TACAGCATGTCCTACTCGCAG | R: GAGGAAGAGGTAACCACAGGG |
| *GAPDH* | F: AGCCACATCGCTCAGACAC | R: GCCCAATACGACCAAATCC |
